# Race/ethnicity moderates the associations between lifetime psilocybin use and opioid use disorder

**DOI:** 10.1371/journal.pone.0321461

**Published:** 2025-05-07

**Authors:** Grant Jones

**Affiliations:** Department of Psychology, Harvard University, Cambridge Massachusetts United States of America; Wingate University, UNITED STATES OF AMERICA

## Abstract

**Background:**

Opioid use disorder (OUD) is a debilitating health condition that is associated with significant morbidity and mortality in the U.S. While preliminary studies have demonstrated that psilocybin is associated with lowered odds of OUD, current research in this domain suffers from a lack of investigation into the impact of race/ethnicity on this association.

**Objective:**

To assess the impact of race and ethnicity on the association between psilocybin use and lowered odds of OUD using data from the National Survey on Drug Use and Health (2002–2019) (N = 706,891).

**Method:**

I used survey-weighted multivariable logistic regression to test whether race/ethnicity moderates the association between psilocybin use and lowered odds of OUD. Subsequently, I stratified my sample by race and ethnicity and assessed the associations between psilocybin and OUD for individual racial and ethnic groups (White, Black, Indigenous, Asian, Multiracial, Hispanic). My analysis plan was pre-registered.

**Results:**

Race and ethnicity significantly moderated the association between psilocybin and OUD. Furthermore, when I stratified my sample by race and ethnicity, only White participants and Hispanic participants demonstrated a link between psilocybin and lowered odds of OUD (White aOR: 0.84; Hispanic aOR: 0.68). For Black, Asian, Indigenous, and Multiracial participants, psilocybin did not share a significant association with OUD.

**Conclusion:**

Race and ethnicity moderate the associations between psilocybin and OUD. Future longitudinal, experimental, and qualitative research is needed to better understand the pattern of associations I observed in this study.

## Introduction

Opioid use disorder (OUD) is a major health issue in the United States [1–4].Opioids contribute to nearly 70% of overdose-related fatalities in America and since the early 2000s [5], deaths related to opioid use disorder have increased by over 300% [6].Classic psychedelics – naturally occurring substances that can give rise to non-ordinary states of consciousness characterized by a sense of awe and transcendence, oneness with the universe, and distortion of time and space – have been linked to the alleviation of various substance use disorders (including OUD) in various cross-sectional, longitudinal, and experimental studies [7–18]. Furthermore, although adverse events related to psychedelics do occur (e.g., extreme anxiety, paranoia), classic psychedelics are generally considered to be safe and have low potential for addiction [19]. Recently, Jones et al., (2022) used population-based survey data to demonstrate that psilocybin – a psychedelic substance that is the active compound in “magic mushrooms” – confers lowered odds of OUD [20]. However, despite this preliminary evidence, there is a severe dearth of investigations into how race and ethnicity may impact the associations that psychedelics share with OUD. The need for such an investigation is timely, as during the coronavirus pandemic, overdose deaths related to opioids sharply increased in communities of color in particular [21, 22]. Therefore, the aim of this study is to investigate how race and ethnicity moderate the association between psilocybin use and lowered odds of OUD using population-based survey data.

Prior research has provided suggestive evidence that psychedelics may have protective benefits against OUD; however, virtually none of these studies assess the impact that race or ethnicity may have on the association between psychedelic use and opioid addiction.

First, Pisano et al., (2017) examined data from the National Survey on Drug Use And Health (NSDUH) (2008–2013) – an annual survey that collects data on substance use and health outcomes in a nationally representative sample of the U.S population – to assess the associations between classic psychedelic use and OUD [12]. Overall, these researchers found that lifetime classic psychedelic use was associated with lowered odds of OUD, even when controlling for a host of demographic factors and lifetime substance use variables. Next, Argento et al. (2022) conducted a longitudinal study consisting of 3813 people who use drugs (PWUD) drawn from a community sample from Vancouver, Canada to assess the impact of psychedelic use on daily prescription opioid use [10]. Similar to Pisano et al. (2017), Argento et al. (2022) found that psychedelic use was associated with 55% reduced odds of daily opioid use.

As previously mentioned, Jones et al. (2022a) attempted to replicate results from Pisano et al. (2017) by using more recent NSDUH data (2015–2019) to examine the associations between psychedelic use and opioid addiction. Additionally, this study also sought to extend the results from Pisano et al. (2017) by examining the associations that individual classic psychedelics (psilocybin, LSD, peyote, and mescaline) share with OUD. Overall, Jones et al. (2022a) replicated and extended the results from Pisano et al. (2017) and demonstrated that psilocybin was the sole classic psychedelic substance to confer lowered odds of OUD in a population-based survey data set [20].

However, as mentioned, a core limitation to all of this existing research is that none of these studies have examined how race and ethnicity might impact these results. Therefore, as many researchers have noted [23, 24], there is a need for additional investigations to address this significant gap within psychedelic research.

The need to investigate the intersection of race and ethnicity, psychedelic use, and OUD is further heightened by prior research indicating that race and ethnicity impact the associations between psychedelic use and lowered odds of various deleterious outcomes. First, Jones et al. (2022b) examined data from the NSDUH (2008–2019) to assess whether race and ethnicity moderate the associations between psilocybin and MDMA/ecstasy use and psychological distress and suicidality, as prior work had demonstrated protective associations between these substances and outcomes =. These researchers found that not only did race and ethnicity moderate the associations between these substances and distress and suicidality, but also that there were fewer and weaker protective associations between psychedelic use and these outcomes for participants of color compared to White participants [25]. Jones (2023) replicated this pattern of results for psilocybin and MDMA use and major depressive disorder (MDE) (i.e., fewer and weaker protective associations between these substances and MDE for participants of color) [26].

Further, Jones et al. (2023) examined whether race and ethnicity moderate the associations between psilocybin use and crime arrests. This study also found race and ethnicity moderates the association between psilocybin and crime, and additionally found protective associations for psilocybin and crime for all racial and ethnic groups except for Black and Hispanic participants, two racial and ethnic groups that are overrepresented in the criminal justice system. Numerous additional studies have found race/ethnicity as well as other identity factors (sexual orientation) may moderate the associations between psychedelic use and health [28–31]. Most recently, Jones et al., 2025 examined a longitudinal sample and found that race/ethnicity moderated the associations between naturalistic psilocybin use and markers related to mental wellbeing (e.g., spiritual well being, cognitive flexibility, emotion regulation) [32]. Overall, these studies paint a preliminary picture that demographic factors and racial and ethnic identity may shape the associations that psychedelic use shares with health outcomes. Consequently, these studies invite further investigation into the ways that race and ethnicity may shape the associations between psychedelic use and opioid addiction.

Thus, the goal of this paper is to use population-based survey data from the NSDUH to investigate how race and ethnicity moderate the associations between lifetime psilocybin use and OUD. This paper focuses on psilocybin use specifically in order to follow up on results from Jones et al. (2022a), which found psilocybin use in particular to be associated with lowered odds of OUD in a NSDUH dataset. I hypothesized that, in line with Jones et al. (2022b) and Jones (2023), there would be fewer and/or weaker protective associations for psilocybin use and OUD for participants of color than there would be for White participants.

## Methods

For this study, I used data from the National Survey on Drug Use and Health (NSDUH); as previously mentioned, the NSDUH is an annual survey dedicated to assessing health outcomes and substance use in a nationally representative sample of the United States population. The NSDUH uses a computer assisted self-interviewing paradigm wherein interviewers administer the survey to participants in their homes; additionally, the NSDUH collects data from U.S. citizens aged 12 years and older in all 50 states as well as in the District of Columbia. I used all available years of NSDUH data for the outcomes of interest (2002–2019) and included all participants aged 18 years and older in the analyses (N *=* 706,891).This study was exempt from review from the Harvard IRB as all data for this study are publicly available and all methods were carried out in accordance with relevant guidelines and regulations.

Independent Variable: Lifetime psilocybin use (yes/no) served as the independent variable within this study, as prior population-based survey research indicated that among classic psychedelics, psilocybin was the specific substance that shared a relationship with lowered odds of OUD [20].

Dependent Variable: The dependent variable was meeting criteria for past year opioid use disorder (OUD). In this study, participants were classified as having OUD if they met DSM-IV criteria (NSDUH years: 2002–2019) for dependence or abuse of prescription pain relievers or heroin in the past year.

Covariates: The following demographic factors and substance use variables served as covariates in this project: sex (male or female), age (18–25, 26–34, 35–49, 50 or older), educational attainment (less than high school, some high school or high school graduate, some college or above), self-reported engagement in risky behavior (never, seldom, sometimes, or always), annual household income (less than $20,000, $20,000–$49,999, $50,000–$74,999, $75,000 or more), marital status (married, divorced/separated, widowed, or never married), survey year (2002–2019), and lifetime use of various substances (MDMA/ecstasy, other classic psychedelics [LSD, peyote, and mescaline], other illegal substances [cocaine, PCP, inhalants] and other commonly misused legal/medicinal substances [tranquilizers, stimulants, sedatives, and marijuana]). I have used these exact covariates in prior population based studies that have assessed the relationship between race/ethnicity, psychedelic use, and deleterious mental health and behavioral outcomes [25–27].

Putative Moderator: I decided a priori to use a three-level race and ethnicity variable as the moderator; this variable was created from the seven-level race and ethnicity variable that is included in the NSDUH survey. I made the decision to reduce the number of levels from seven to three in order to optimize statistical parsimony and reduce the number of interaction tests in this study. Additionally, I have used this three-level race and ethnicity moderator in all of my prior population-based studies of race/ethnicity as a moderator of the link between psychedelic use and deleterious outcomes [25–27], allowing for comparability between the results of this study and my prior work.

The three levels of my moderator are: Non Hispanic White (N = 446,937), Non-Hispanic Participant of Color (N = 148,815), and Hispanic (N = 111,139). The Non-Hispanic Participant of Color category includes individuals from the following racial/ethnic groups: Non-Hispanic Black (N = 86,970), Non-Hispanic Native American/Alaska Native (N = 10,132), Non-Hispanic Native Hawaiian/Pacific Islander (N = 3,514), Non-Hispanic Asian (N = 28,535), and Multiracial (N = 19,664) individuals.

Analysis: I conducted all analyses in R using the ‘Survey’ package, as this software allows one to incorporate the survey weights and complex survey design of population-based surveys like the NSDUH into one’s analyses. The analysis plan consists of two steps, and this plan was pre-registered on the Open Science Framework (https://osf.io/yk42j). The analysis plan was updated following registration; these changes are detailed in the aforementioned OSF link. Additionally, these steps are in line with prior population-based survey research on race/ethnicity, psychedelic use, and health outcomes.

First, I used survey-weighted logistic regression to assess whether race and ethnicity moderates the association between psilocybin and OUD. This step served as a preliminary test of whether there are significant differences between White participants versus participants of color in the associations that psilocybin shares with opioid addiction. Therefore, I did not interpret the specific beta values that would be yielded by these moderation tests, but simply assessed whether or not these tests were significant; if I received a significant result for either of the two contrasts within my interaction test, I would use these results to justify proceeding to the next step of my analyses.

In step two of my analysis plan, I separated my sample based on racial and ethnic identity and used survey-weighted logistic regression to assess the associations that psilocybin use shares with OUD for each racial and ethnic group. I separated my sample into six groups based on race and ethnicity: Non-Hispanic White, Black, Indigenous, Asian, Multiracial, and Hispanic participants. The Indigenous category includes individuals who identify as Non-Hispanic Native American/Alaska Native or as Non-Hispanic Native Hawaiian/Pacific Islander. I combined these two groups as the relatively small sample sizes of these two groups can cause errors when attempting to conduct survey-weighted logistic regression; I have taken this approach in my prior work as well [25–27].

## Results

The total sample size for this study was N = 706,891. Tables 1 and 2 presents the demographics of my sample as well as rates of psilocybin use and OUD, stratified by race and ethnicity; Table 1 details this information as weighted percentages, whereas Table 2 does so as unweighted counts. Psilocybin use was most prevalent in Non-Hispanic White and Multiracial participants, whereas OUD was most prevalent in Native American/Alaska Native and Multiracial participants. In addition, Multiracial and Hispanic participants featured a significantly greater share of younger participants compared to other racial/ethnic groups. Lastly, Black and Asian participants were most likely to endorse never engaging in risky behavior.

**Table 1:** Participant demographics (weighted %).

Characteristic	Non-Hispanic White (weighted %)	Non-Hispanic Black (weighted %)	Non-Hispanic Native American/Alaska Native (weighted %)	Non-Hispanic Native Hawaiian/Pacific Islander (weighted %)	Non-Hispanic Asian (weighted %)	Non-Hispanic Multiracial (weighted %)	Hispanic (weighted %)	P- value^1^
Lifetime Psilocybin Use	11.6%	1.7%	10.6%	6.2%	2.7%	14.7%	5.0%	<0.001
Marital Status								<0.001
Married	57.6%	33.5%	40.7%	51.1%	62.6%	40.1%	50.1%	
Widowed	6.8%	6.3%	6.3%	3.0%	3.5%	6.7%	3.4%	
Divorced or Separated	13.5%	17.7%	18.7%	11.5%	6.5%	18.1%	12.8%	
Never Been Married	22.1%	42.5%	34.3%	34.5%	27.4%	35.0%	33.6%	
Yearly Household Income								<0.001
<$20,000	14.1%	31.6%	35.7%	21.0%	13.5%	24.8%	25.6%	
$20,000-$49,999	30.6%	36.7%	37.2%	34.8%	24.2%	34.2%	41.1%	
$50,000-$74,999	18.4%	13.9%	13.1%	16.6%	16.5%	15.5%	14.1%	
$75,000+	36.9%	17.8%	14.0%	27.6%	45.7%	25.4%	19.2%	
Age								<0.001
18–25	12.6%	17.5%	17.4%	19.0%	15.7%	19.4%	20.0%	
26–34	13.9%	17.5%	17.0%	19.5%	21.1%	17.2%	22.4%	
35–49	25.9%	28.4%	27.5%	30.5%	31.7%	22.7%	30.8%	
50+	47.6%	36.5%	38.1%	31.0%	31.5%	40.7%	26.8%	
Educational Attainment								<0.001
Less than HS	2.4%	3.6%	5.5%	3.8%	2.2%	3.2%	18.0%	
Some HS/HS Grad	37.2%	49.2%	53.7%	45.3%	17.7%	40.8%	44.4%	
Some College or Above	60.4%	47.2%	40.9%	50.9%	80.1%	56.0%	37.6%	
Self-Reported Engagement in Risky Behavior								<0.001
Never	46.0%	67.8%	55.2%	57.5%	63.4%	46.0%	66.3%	
Seldom	38.6%	22.3%	29.5%	26.2%	26.8%	37.1%	22.6%	
Sometimes	14.1%	8.8%	13.4%	14.3%	8.6%	14.9%	9.6%	
Always	1.3%	1.0%	1.9%	2.1%	1.2%	2.0%	1.5%	
Opioid Misuse	0.8%	0.7%	1.4%	0.8%	0.3%	1.1%	0.7%	<0.001

^1^chi-squared test with Rao & Scott’s second-order correction

**Table 2:** Participant demographics (unweighted counts).

Characteristic	Non-Hispanic White (unweighted count)	Non-Hispanic Black (unweighted count)	Non-Hispanic Native American/Alaska Native (unweighted count)	Non-Hispanic Native Hawaiian/Pacific Islander (unweighted count)	Non-Hispanic Asian (unweighted count)	Non-Hispanic Multiracial (unweighted count)	Hispanic (unweighted count)	p-value^1^
Lifetime Psilocybin Use	62,170	1,519	1,229	245	1,034	2,996	6,548	<0.001
Marital Status								<0.001
Married	191,877	18,525	2,927	1,181	12,432	5,303	40,228	
Widowed	13,984	2,389	248	53	390	440	1,622	
Divorced or Separated	45,346	9,317	1,235	246	1,151	2,177	9,819	
Never Been Married	195,730	56,739	5,722	2,034	14,562	11,744	59,470	
Yearly Household Income								<0.001
<$20,000	87,593	32,541	3,859	962	6,024	5,548	32,188	
$20,000–$49,999	142,546	32,125	3,803	1,303	7,553	6,945	47,212	
$50,000–$74,999	78,484	10,423	1,239	527	4,457	2,924	14,776	
$75,000+	138,314	11,881	1,231	722	10,501	4,247	16,963	
Age								<0.001
18–25	179,344	41,086	4,661	1,717	12,478	10,171	55,282	
26–34	71,322	14,691	1,772	657	5,989	3,318	21,818	
35–49	106,064	19,051	2,360	766	6,874	3,731	23,783	
50+	90,207	12,142	1,339	374	3,194	2,444	10,256	
Educational Attainment								<0.001
Less than HS	7,853	1,599	328	72	404	357	13,716	
Some HS/HS Grad	178,086	46,353	6,193	1,959	6,369	9,167	56,743	
Some College or Above	260,998	39,018	3,611	1,483	21,762	10,140	40,680	
Self-Reported Engagement in Risky Behavior								<0.001
Never	166,770	53,646	4,724	1,742	15,170	7,324	64,855	
Seldom	184,383	22,032	3,203	990	8,870	7,564	28,791	
Sometimes	84,730	9,679	1,824	634	3,704	4,067	14,511	
Always	10,328	1,262	331	118	440	661	2,341	
Unknown	294,294	110,051	6,377	2,725	146,583	12,744	192,577	
Opioid Misuse	6,016	614	180	43	101	321	970	<0.001

^1^chi-squared test with Rao & Scott’s second-order correction

Table 3 presents the results of my interaction tests between race/ethnicity and lifetime psilocybin use and the association between these interactions and OUD. In line with my hypothesis, the interaction between the “Non-Hispanic Participant of Color” category and psilocybin use was significantly associated with OUD (beta = -0.40; 95% Confidence Interval: -0.65, -0.15; p < 0.01). The “Hispanic” category did not interact with psilocybin use. Given that one of my contrasts was significant, I proceeded with stratifying my sample by race and ethnicity and assessing the associations that psilocybin shares with OUD for each racial and ethnic group.

**Table 3:** Interaction between race/ethnicity and psilocybin use for predicting opioid use disorder.

Interaction Test	beta (95% CI)^12^	p-value
Race/Ethnicity * Lifetime Psilocybin Use		
Non-Hispanic Person of Color * Lifetime Psilocybin Use	-0.40^**^ **(-0.65, -0.15)**	0.002
Hispanic * Lifetime Psilocybin Use	-0.10 (-0.36, 0.17)	0.5

^1^*p<0.05;

**p<0.01; CI = confidence interval; Reference category: White participants

Table 4 presents the results of my stratified analyses assessing the relationships between psilocybin and OUD for each racial and ethnic group. For White and Hispanic participants, psilocybin conferred lowered odds of OUD (White aOR: 0.84; 95% CI: 0.76, 0.94; p < 0.01) (Hispanic aOR: 0.68; 95% CI: 0.47, 0.97; p < 0.05)). For all participants included in the “Non-Hispanic Participant of Color” group (Black, Indigenous, Asian, Multiracial), psilocybin did not share significant associations with OUD. Overall, these findings are largely in line with my hypothesis, which posited that there would be fewer and/or weaker associations between psilocybin and OUD for people of color in this study.

**Table 4:** Associations between psilocybin and opioid use disorder, stratified by race and ethnicity.

Group	aOR (95% CI)^12^	p-value
White	0.84^**^ (0.76, 0.94)	0.002
Black	0.86 (0.51, 1.45)	0.6
Indigenous	0.86 (0.47, 1.58)	0.6
Asian	0.62 (0.22, 1.74)	0.4
Multiracial	0.67 (0.36, 1.24)	0.2
Hispanic	0.68^*^ (0.47, 0.97)	0.033

*p<0.05;

**p<0.01; aOR = adjusted odds ratio; CI = confidence interval

## Discussion

The goal of this study was to examine whether race and ethnicity moderates the association between lifetime psilocybin use and lowered odds of OUD in a population-based survey sample from the National Survey on Drug Use and Health. Overall, I found that there was a significant interaction between a “Non-Hispanic Participant of Color” identifier and psilocybin use that was associated with OUD. Furthermore, when I examined the associations between lifetime psilocybin use and OUD for each racial and ethnic group, I found that psilocybin conferred lowered odds of OUD only for White and Hispanic participants. For all groups included in the “Non-Hispanic Participant of Color” category (Black, Indigenous, Multiracial, Asian), psilocybin did not have a significant association with OUD. This study is cross-sectional and uses lifetime use variables in all analyses; these limitations sharply limit the ability to derive causal conclusions from the results. Nevertheless, these findings provide preliminary evidence that racial and ethnic identity may impact the associations between psilocybin use and lowered odds of opioid addiction.

### Potential explanations

There are a few potential explanations for my findings in this study, many of which overlap with explanations that I have provided in my prior research within this domain [25–27].

**Demographic differences in motivations for and rates of psychedelic use**. First, there may be demographic differences in rates and motivations for psilocybin use that underlie my findings in this study. For instance, it is known that Indigenous communities have used classic psychedelics in worshiping ceremonies for centuries [33]. While this specific cultural relationship to psychedelic use may not have shaped the findings in this study, it nevertheless illustrates the ways that individual racial and ethnic groups may have unique motivations for using psychedelics that may have shaped my results. The possibility that different racial and ethnic groups may use psychedelics for differing reasons is mirrored by the fact that there are differing rates of psilocybin use across racial and ethnic groups (see Tables 1 and 2), a finding that has been documented in other epidemiological studies of psilocybin as well [34]. Such differences in motivations for use may drive the differing associations observed in this study for White and Hispanic participants versus Non-Hispanic Participants of Color.

Although differing rates and motivations for psychedelic use may have driven the findings in this study, there is a severe dearth of research on the motivations behind psychedelic use in communities of color. Therefore, future studies should further investigate this research domain. Such investigations can also further elucidate why I found a significant association between psilocybin and lowered odds of OUD for Hispanic participants in this study, as Hispanic participants were the only participants of color for whom I found this association. The “Hispanic” community is a diverse group consisting of individuals from a range of racial and socioeconomic backgrounds. Accordingly, there may be a sub-demographic of Hispanic individuals who use psilocybin at greater rates and also simultaneously have lowered odds of OUD, leading to my finding for Hispanic participants within this study. Additional research that examines sub-demographic differences of psilocybin use in racial and ethnic groups can shed further light on my results as well.

**The impact of “set and setting” on psychedelic use in communities of color.** Another potential explanation for my findings is the impact of “set and setting” of the effects of psychedelic use in communities of color. The term “set and setting” refers to the notion that both one’s mind “set” and the “setting” in which one takes psychedelics have a strong impact on the nature of one’s psychedelic experience [35]. The “Non-Hispanic Participants of Color” in this study were likely taking psilocybin in the American “setting,” in which racism and discrimination are pervasive [36–40]. Relatedly, this fact means that illegal psilocybin use is riskier for these participants, as illegal substance use can be more likely to result in incarceration for individuals of color [41]. Downstream, the inbuilt stressors of racism and discrimination may mean that the potentially salutary elements of a psychedelic experience – such as mystical experience and awe [42, 43] – may occur less frequently for participants of color who use psilocybin. Future studies should investigate the impact of racism and discrimination on the psychedelic experience for individuals of color, as these investigations can shed further light on this potential driver of my results.

**Racial and ethnic disparities in OUD treatment and outcomes.** The last potential explanation for my findings is that racial and ethnic disparities in OUD treatment and outcomes may make it less likely for psilocybin to confer lowered odds of OUD. A core mechanism via which psilocybin and other classic psychedelics are theorized to confer salutary benefits is due to positive behavioral changes (i.e., increased meditation practice, finding support groups or counseling for mental health issues) that take place following the psychedelic experience [44]. Furthermore, in experimental and observational studies in which classic psychedelics were associated with reductions in substance misuse, participants simultaneously reported positive behavioral changes that coincided with these reductions [45–47]. Thus, psilocybin may promote lowered odds of OUD by eliciting positive behavioral shifts and treatment seeking behaviors in those who use it.

Yet, individuals of color may face structural barriers to making positive behavioral changes or achieving positive outcomes related to treatment and recovery from OUD, which may make it less likely for psilocybin use to confer lowered odds of OUD in these populations. For example, Entress (2021) analyzed data from the Treatment Episode Data Set Discharges (TED-S) – a dataset that collects information on annual discharges from substance use treatment facilities – to assess racial and ethnic disparities in OUD treatment [48].This researcher found individuals of color have a lower likelihood of being treated for OUD using medication (a gold standard approach for treating OUD), being referred to treatment for OUD by a medical professional, and leaving treatment due to the treatment course being completed (i.e., instead, leaving against medical advice, being terminated from treatment, death during treatment, etc). Finally, a 2016 cross-sectional study by Wu et al. assessed utilization of OUD treatment services and found that many communities of color – including Black, Native Hawaiian/Pacific Islander, and Asian communities – underutilized OUD treatment compared to White individuals [49]. Such disparities may indicate that there are additional hurdles that individuals of color may face in attempting to make positive choices related to their opioid misuse, a fact that may have contributed to the results within this study. Future research that navigates the intersection of structural inequalities in OUD-related care, naturalistic psychedelic use, and OUD outcomes can elucidate the validity of this potential explanation.

### Limitations + future directions

There are a few important limitations to this work. As mentioned in the first paragraph of the Discussion, the first and most important limitation is that this study featured cross-sectional data, meaning that this study cannot be used to make causal claims about the impact of race and ethnicity on psychedelic use and lowered odds of OUD. Future longitudinal and experimental studies will be needed to further assess causality within this research domain.

Next, due to limitations of the NSDUH data set, I used a binary, lifetime use variable to assess psilocybin use in this study. However, as a result, that means that I could not assess frequency or recency of psilocybin use, representing another major limitation to my work. Future investigations that can assess psilocybin use in a more granular fashion will be able to shed even more light on my results.

A third limitation to my study is driven by the exclusion criteria of the NSDUH survey. The NSDUH does not sample from individuals experiencing homelessness that do not reside in shelters, active duty military members, or individuals living in institutionalized group quarters (e.g., hospitals, prisons, nursing homes). Some of these populations may use opioids at higher rates, therefore limiting the generalizability of my study. Future studies should assess whether the pattern of results observed in this study – in which there were no protective associations between psilocybin and OUD for Non-Hispanic Participants of Color – applies to populations that are currently excluded from the NSDUH; downstream, such investigations may inform investigations into using psychedelics as treatments for OUD in these populations.

Fourth, the racial and ethnic categories included in the NSDUH dataset are limited, preventing more nuanced investigations into the impact of identity on the link between psilocybin use and OUD. The NSDUH variable only features seven categories for one to identify their racial or ethnic identity, greatly restricting more granular assessments on the interplay between race/ethnicity, psilocybin, and OUD. Future research that allows for participants to self-identify in more expansive and flexible ways can give rise to greater insight into this research area.

Finally, the last limitation to my study is that some of the point estimates of the adjusted odds ratios (aOR) for Non-Hispanic Participants of Color overlap with those of White and Hispanic participants. For instance, for White participants, the aOR for psilocybin and OUD was 0.84; for Black participants, the aOR was 0.86, but this association was not significant in my study. Further, although none of the associations between psilocybin and OUD for Non-Hispanic Participants of Color were significant, these overlapping aORs nevertheless raise questions of whether there are truly differences between these groups in my study. However, I do not believe this limitation significantly hinders my findings. The interaction tests clearly indicated that Non-Hispanic Participants of Color feature differing associations between psilocybin and OUD compared to White participants. Additionally, given the large sample of over 700,000 participants in this study, each individual racial and ethnic group in this study also features a robust sample size for my logistic regression models, allowing one to detect significant associations between psilocybin and OUD for all racial and ethnic groups. Future replication studies, longitudinal studies, and qualitative investigations can instill even greater confidence in these results.

## Conclusion

The goal of this study was to assess whether race and ethnicity moderates the association between psilocybin use and lowered odds of OUD in a cross-sectional, population-based survey dataset. Overall, I found that “Non-Hispanic Participants of Color” featured differing associations between psilocybin and OUD compared to White participants. When I further assessed these associations for individual racial and ethnic groups, I found that only White and Hispanic populations featured protective associations between psilocybin use and OUD; these protective associations did not exist for any Non-Hispanic Participants of Color. My findings may be driven by demographic differences in rates of and motivations for psychedelic use, the impact of “set and setting” on the effects of psychedelic use in communities of color, and structural disparities in OUD treatment and recovery. Future longitudinal and experimental studies can shed further light on the findings in this study. Overall, this study represents incremental progress in better understanding the impact of identity on the potential protective associations between psychedelics and disordered opioid use.
